# Making Natural Products Supernatural

**DOI:** 10.1021/acscentsci.4c00695

**Published:** 2024-05-14

**Authors:** Alla Katsnelson

Picrotoxinin is a compound with a far-reaching history. Very bitter and highly toxic, it occurs naturally in the seeds of a climbing plant called *Anamirta cocculus*, which grows in Southeast Asia. For centuries, fishers have sprinkled the crushed and powdered seeds into bodies of water to stun fish, making them easier to catch. And practitioners of several different systems of medicine have long used these seeds both topically and internally to treat a wide range of ailments, including motion sickness, scabies, rheumatism, and nervous system conditions.

**Figure:**
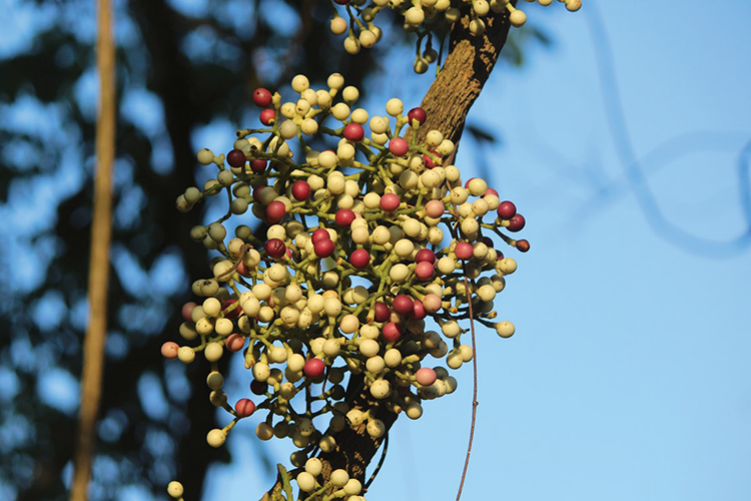
The fruits of *Anamirta cocculus* contain picrotoxinin, a paralysis-inducing compound. Ryan Shenvi’s lab synthesized an analog of the molecule that is more stable than its natural counterpart and has different binding properties. Credit: RAJENDRANTM/Wikimedia Commons.

But efforts to bring picrotoxinin into the practice of Western medicine have so far been a bust. In the plant, the compound exists as a 1:1 mixture with a related, less-active molecule, picrotin. Chemists long struggled to separate them, only to find that picrotoxinin is highly complex, unstable, and chemically puzzling. For decades, chemists could not crack its structure or tame its toxicity, and they found its instability confounding. Today picrotoxinin (mixed with picrotin) is used mostly as a research tool for studying a type of ion channel receptor for the neurotransmitter γ-aminobutyric acid, or GABA, because it selectively blocks these receptors' function.

Picrotoxinin’s GABA-receptor-hobbling capability is what first drew Ryan Shenvi to the compound. Shenvi, a synthetic chemist at Scripps Research, was searching for compounds that target ion channels in the brain, a quality that might potentially be useful in developing new therapies. He thought picrotoxinin’s long history offered a solid foundation.

Shenvi and his colleagues developed an efficient method for synthesizing the compound, which involved embedding a methyl group at the bridge lactone position. Late last year, they reported that the modification, originally used transiently to make the compound easier to build, also made it more stable. What’s more, it changed the compound's selectivity to a related but different ion channel, pointing to how exactly structural changes affect the molecule’s function.

“We realized that we can make a very close analog [to picrotoxinin] that might actually be superior,” Shenvi says, with a yield 10 times as high as that of the original version. In general, he explains, his lab is not so much in the business of making natural products but in making what he and others have begun to call supernatural products. “We try to make something that’s almost identical but that’s predicted to be better [than natural products]—not to mimic their structure and function but actually to surpass them.”

This philosophy is driving a transformation in natural product synthesis. Since the field’s emergence in the early 20th century, researchers have poured enormous efforts into developing lab techniques to enable total synthesis of these large and complex organic compounds that in nature are synthesized by living organisms. But the broad capabilities to riff on these compounds—that is, to systematically alter them to hone their function—have been absent. Now those capabilities are emerging.

“People have been dreaming of this for decades, but the chemistry hasn’t been there,” says Seth Herzon, an organic chemist at Yale University. “What’s exciting in the current landscape is that the synthetic chemistry is catching up very rapidly with peoples’ ambitions in terms of being able to make these modifications to natural products.”

That is good news for the field. “‘Renaissance’ doesn’t even feel strong enough,” says Martin D. Burke, a chemist at the University of Illinois Urbana-Champaign. “I think we are about to have an absolute explosion of really exciting natural products research because new tools are now in hand.”

## Tools get better, chemists get closer

Compounds that chemists generally refer to as natural products are mainly secondary metabolites produced by living organisms—plants, bacteria, fungi, animals, and protists. These substances evolved over millions of years to perform specific functions, such as making an organism less palatable to its predators or in some other way improving its odds for survival. People have harnessed these chemicals for medicinal, spiritual, agricultural, or other uses for millennia.

These compounds have also been crucial in drug development. By some estimates, today half of all medicines approved by the U.S. Food and Drug Administration have taken their inspiration from natural products. The fact that these molecules are bioactive by design gives them an edge as potential drugs. “They grew up in a biological milieu, so they get around in organisms,” says Dale Boger, a chemist at Scripps Research.

But because these molecules are specialized to act in other organisms, their biological activity often does not match up to our medicinal needs, and chemists have long sought to create analogs with more finely tuned functions. In the past, researchers began by first synthesizing the natural product itself—which wasn’t always straightforward. Relying on natural products as starting materials “has been fruitful, and it’s given us a lot of structures,” says Herzon, “but it’s very constrained because the molecule is setting the boundary conditions on the type of chemistry people are carrying out.”

Sweeping advances in both understanding and technology are enabling chemists to pursue more innovative syntheses and, increasingly, to bypass that part of the process. One key element is the ability to determine structure.

Techniques such as microcrystal electron diffraction allow chemists to literally see how a natural product interacts with a target protein. The method also can give a better sense of how tweaking the natural product’s structure can enhance that interaction.

Plug-and-play molecular biology tools, which are used for DNA and RNA sequencing and proteomics, make it possible for natural product chemists to work at the interface of biology by probing the activity of the molecules they make. “You can now really get a complete picture of a fully synthetic natural product—what it does on a genetic level, what it does on a proteomic level—and you can do that quickly, easily, and reliably in a way you never could before,” says James Frederich, a chemist at Florida State University. That means chemists can, in their own laboratories, do a lot of preliminary characterization of compounds they synthesize, quickly discarding ones that do not pass muster on functional grounds.

But perhaps the biggest driver has been the growing sophistication of synthesis. One major turning point came more than 15 years ago, when M. Christina White at the University of Illinois Urbana–Champaign and her team developed the first methods for carbon–hydrogen bond activation.

At the time, chemists dismissed the possibility of swooping into an already existing structure to oxidize a late-stage C–H bond. But White recognized that such switcheroos routinely occur in nature. She set out to mimic them by developing small-molecule catalysts that can selectively target features such as steric versus stereoelectronic or electronic effects on different C–H bonds. “We are not yet very good at connecting structure to function, but one thing we do know is that these atomistic changes can dramatically affect function,” White says.

Other chemists have since added to the synthetic chemistry toolbox, providing a path to exploring the connection between structure and function in a systematic way. One example is the development of reactions that enable skeletal editing—making highly specific changes to a compound’s structure close to its core—although the approach is still in its infancy, says Richmond Sarpong, a chemist at the University of California, Berkeley.

**Figure:**
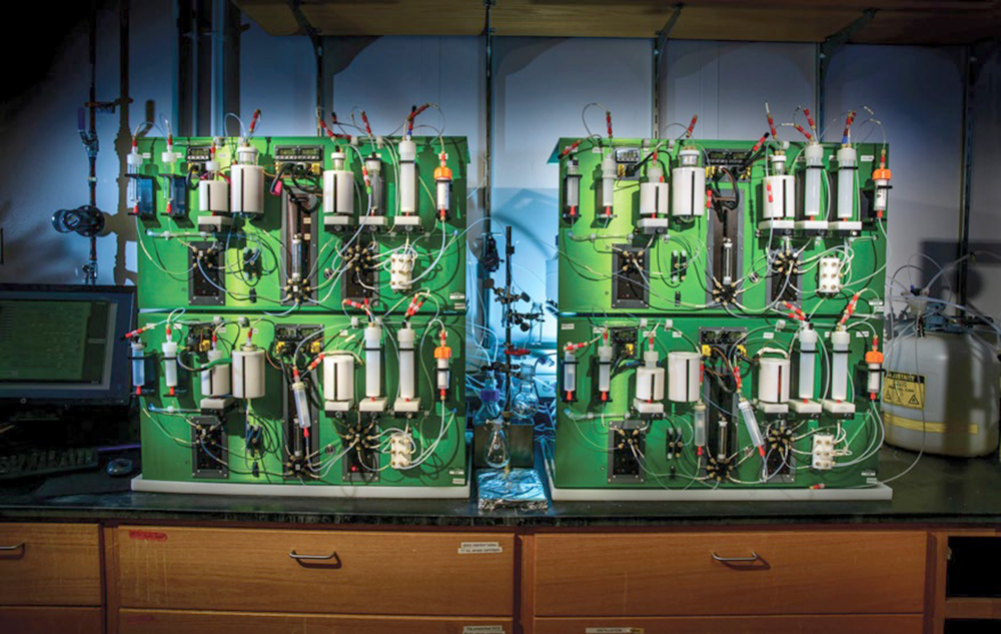
Martin Burke and his team created an automated small-molecule maker that can synthesize many types of 3D molecules. Credit: UI News Bureau/Fred Zwicky.

That work in turn is helping synthetic chemists take a modular approach to improving natural products. Burke’s team, for example, created a robot that can automate chemical synthesis to build increasingly complex organic molecules. Their work and others’ work suggest that chemists can imitate nature’s way of producing secondary metabolites using just a handful of building blocks and reactions that researchers can now draw on. “The capacity to build natural products in a modular, automated fashion will revolutionize our capacity to extract their functional potential,” Burke says.

## Supernatural steps forward

As these capabilities add up, chemists are able to be more ambitious in their synthetic planning and to explore variations in the chemical structures in ways that were not previously conceivable.

In an example of such work published earlier this year, Andrew Myers at Harvard University and his colleagues reported a synthetic antibiotic called cresomycin inspired by clindamycin, which was itself a derivative of a naturally occurring antibiotic.

Cresomycin is designed with an extra-strength ability to bind to ribosomes of many different types of bacteria and disrupt their function. The strength with which this new molecule binds, combined with its rigidity, circumvents the bacteria’s propensity to develop antibiotic resistance. The team synthesized it by first building its components and then assembling them, like a Lego model. “Holy cow, it’s like a total redo” from a previous version, Herzon says. “It’s like turning a Volkswagen into a Porsche.”

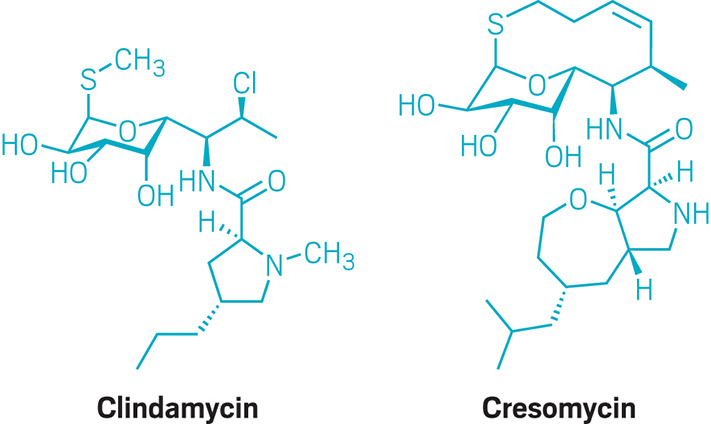



Herzon’s lab is taking this approach with a compound called pleuromutilin, which was isolated from a fungus in the 1950s and has antibiotic activity. The molecule is a terpene, derived from five-carbon building blocks. Because it is essentially a massive hydrocarbon, the methods that are available to improve it starting with its own structure are limited, Herzon says, so his team has been trying to build it out from scratch to create an antibiotic with clinical potential. “The idea is to engineer into our synthetic planning some changes that we think might be beneficial from a biological standpoint,” he says.

Boger’s work on an antibiotic called vancomycin, which he began about three decades ago, reflects how natural product synthesis has progressed. Originally isolated from soil bacteria, vancomycin was first used clinically in 1955 and is today considered a crucial antibiotic of last resort for hard-to-treat infections such as those caused by methicillin-resistant *Staphylococcus aureus* (MRSA). But its power is threatened by increasing bacterial resistance, which emerged in the mid-1980s.

**Figure:**
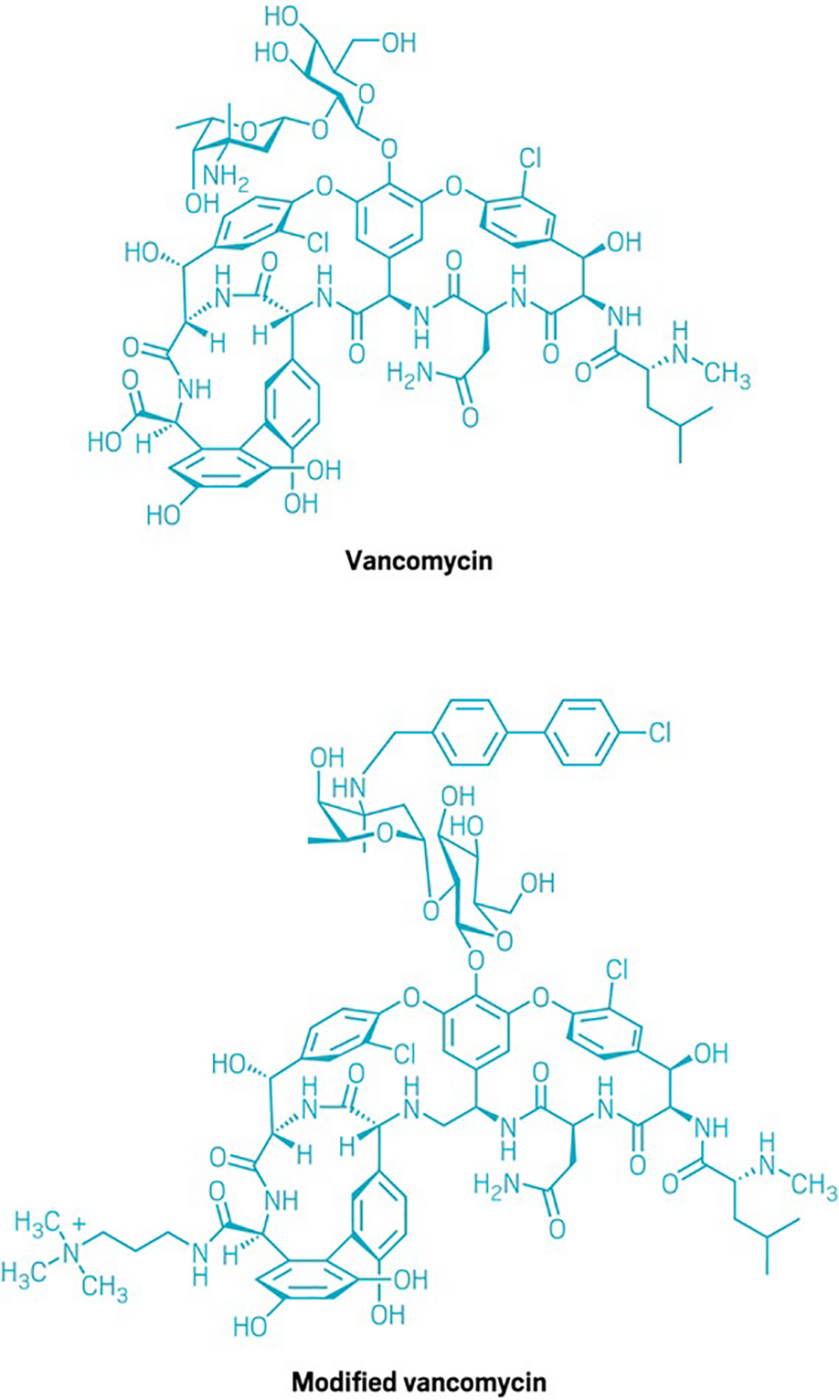
After multiple iterations, Dale Boger and his colleagues created a version of vancomycin that acts by three independent mechanisms of action. The researchers’ modifications enable the molecule to interfere with key resistance mechanisms in bacteria and dramatically boost the antibiotic’s potency.

The compound is manufactured by fermentation but Boger was one of three chemists to independently synthesize it in the late 1990s, and he and others have been working since then to create an analog that its bacterial targets cannot evade. After multiple iterations, he and his colleagues created a version of the compound with three alterations. They figured out that removing a single oxygen atom at the molecule’s core would allow it to interfere with the resistance mechanism in bacteria, reviving the microorganisms’ sensitivity to the drug. They also brought in two changes at the molecule’s periphery that added two new mechanisms of action.

The souped-up vancomycin “now acts by three independent mechanisms of action—and bacteria struggle to work around all three simultaneously,” Boger says. In addition to making it harder for resistance to develop anew, the changes boost vancomycin’s potency 1,000-fold.

Boger’s team also developed a pared-down synthesis for the molecule that could make its clinical production viable. Many of the synthetic methodologies Boger’s team used in the work—including some reactions as well as strategies they used to plan the antibiotic synthesis—didn’t exist 25 years ago. And undoubtedly, he says, methods for making more innovative compounds like it will continue to advance. “The changes we made couldn’t be done in any other way today,” he says. But “I think someday they will be.”

*Alla Katsnelson is a freelance contributor to*Chemical & Engineering News*, the independent news outlet of the American Chemical Society.*

